# Effect of Perceived Stress on Sleep Procrastination in College Students: A Chain-Mediated Role Between Depression and Inhibitory Control

**DOI:** 10.3390/bs16081419

**Published:** 2026-08-18

**Authors:** Ran Ke, Xin Miao, Chundi Peng, Fei Yu, Dong Han, Ruobing Zhang, Weiye Chen, Ruyi Li, Xinyao Liu, Zhengxue Qiao, Yanjie Yang

**Affiliations:** 1 Psychology and Health Management Center, Harbin Medical University, Harbin 150088, China; keran97@126.com (R.K.); 17863257008@163.com (X.M.); 17863867853@163.com (F.Y.); gloria2430248720@163.com (R.Z.); 15603600195@163.com (W.C.); moguxrlivia@gmail.com (R.L.); l1921499843@163.com (X.L.); 2 The School of Mental Health, Wenzhou Medical University, Wenzhou 325015, China; pcd2631048711@163.com; 3 Department of Human Movement and Sport Science, Harbin Sport University, Harbin 150096, China; handong@hrbipe.edu.cn

**Keywords:** sleep procrastination, perceived stress, depression, inhibitory control, college student

## Abstract

Background: Sleep procrastination is a prevalent health behavior problem among college students, yet the psychological mechanisms through which perceived stress influences this behavior remain incompletely understood. Drawing upon Temporal Self-Regulation Theory, this study aimed to explore the relationship between perceived stress and sleep procrastination among college students with a particular focus on the serial mediating roles of depression and inhibitory control. Results: The mean sleep procrastination score was 37.20 ± 3.88. Perceived stress was significantly positively correlated with sleep procrastination (r = 0.596, *p* < 0.01) and depression (r = 0.605, *p* < 0.01) and negatively correlated with inhibitory control (r = −0.520, *p* < 0.01). Mediation analyses revealed that depression (indirect effect = 0.180, 95% confidence interval [CI] [0.147, 0.215]) and inhibitory control (indirect effect = 0.042, 95% CI [0.021, 0.064]) independently mediated the relationship between perceived stress and sleep procrastination. Moreover, a significant serial mediation pathway through depression followed by inhibitory control was identified (indirect effect = 0.014, 95% CI [0.007, 0.023]). Conclusions: These findings indicated that perceived stress is associated with sleep procrastination both directly and indirectly through sequential associations involving depression and inhibitory control. Specifically, higher perceived stress corresponded to greater depressive symptoms, which in turn were related to reduced inhibitory control, and this chain of associations is further linked to increased sleep procrastination. This study provides a novel theoretical contribution by an associative chain mediation mechanism underlying sleep procrastination. Practical implications include the potential value of stress reduction interventions, depression management, and inhibitory control training as strategies to mitigate sleep procrastination among college students.

## 1. Introduction

Sleep procrastination is the inability of an individual to go to bed at a predetermined time without external obstacles (Kroese et al., 2014). In the past decade, the average daily sleep duration of Chinese people has shown a trend of decreasing year after year (i.e., a delay in falling asleep by 2 h and a decrease in total sleep duration of 1.5 h), according to the China Sleep Research Report released in 2022. This phenomenon is regarded as a typical health behavior problem, especially among college students (Huang et al., 2023; Zhu et al., 2022). College students, as a group at a key stage of mental development, frequently face multiple pressures and challenges, such as academic burdens and interpersonal relationships, which often contribute to the accumulation of stress and then induce or aggravate sleep procrastination behaviors. Studies have shown that up to 65.7% of college students exhibit varying degrees of sleep procrastination (Huang et al., 2023). This phenomenon will not only lead to a decline in sleep quality and sleep duration in the short term, but also lead to an increase in daytime fatigue, which will adversely affect academic performance and daily life. In the long term, persistent sleep procrastination may also cause profound and irreversible damage to the physical and mental health of college students (Herzog-Krzywoszanska et al., 2021). Therefore, an in-depth discussion and scientific research on the problem of college students’ sleep procrastination are urgently needed.

Intention, behavioral pre-potency (the likelihood of behavior considering habit and environmental cues), and self-regulatory capacity (an individual’s capacity to exert control over themselves) directly influence behavior, with behavioral pre-potency and self-regulatory capacity also moderating the intention-behavior relationship, according to temporal self-regulation theory (TST) (Hall & Fong, 2007). TST provides a framework for analyzing the mechanisms underlying perceived stress, depression, and inhibitory control affecting sleep procrastination in college students. Within the TST framework, perceived stress can be conceptualized as a factor that weakens sleep-related intentions through cognitive bias and emotional depletion, while simultaneously undermining the translation of these intentions into actual sleep behaviors. Depression, as a negative affective state, interferes with the cognitive appraisal of sleep importance and the self-regulatory processes necessary for timely sleep onset. Inhibitory control, which aligns closely with the self-regulatory capacity component of TST, represents an individual’s ability to override prepotent responses, such as engaging in enjoyable but sleep-disrupting activities, in favor of goal-directed sleep behaviors. The serial mediation model proposed in this study is theoretically grounded. Specifically, perceived stress is expected to exacerbate depressive symptoms (affecting intention formation), which in turn impairs inhibitory control (undermining self-regulatory capacity) and collectively contributes to sleep procrastination (behavioral outcome). This conceptualization is consistent with the TST premise that intention-related and self-regulatory mechanisms are critical in shaping health behaviors.

Perceived stress is associated with weakened sleep motivation through cognitive bias and emotional exhaustion, interferes with the formation of intention, and is associated with the preference for instant gratification and resource depletion that impede the conversion of intention into behavior. Depression is associated with distorted cognition of sleep importance cognitively, causes an imbalance of time-bound titer, and forms a bad behavioral pattern that interferes with the execution of intention. The inhibitory control defect makes it difficult for the individual to resist the temptation before going to bed, emotion management ability decreases, and intention conversion is hindered. The following section will detail how these three factors jointly affect sleep procrastination in the stages of intention formation and transformation through the interaction of the TST framework (Dorina et al., 2024).

Perceived stress refers to an individual’s subjective cognition and evaluation of stressful events, which originates from the dynamic interaction process between the individual and the environment (Lazarus & Folkman, 1984). It is worth noting that high pressure is often accompanied by an increased risk of procrastination, which can be regarded as a strategic avoidance mechanism in the face of pressure (Sirois et al., 2023). Individuals are more inclined to choose procrastination behavior to relieve psychological pressure when faced with high-intensity perceived pressure (Bernecker & Job, 2020). Some studies have revealed that there is a positive correlation between an individual’s perceived level of stress and the severity of sleep procrastination, i.e., the higher the perceived level of stress, the more prominent the individual’s sleep procrastination phenomenon will be (Chi et al., 2021; Schmidt et al., 2024).

Depression is a persistent negative mood with symptoms including long-term low mood and lack of lasting and stable pleasure (K. R. Cohen & Peachey, 2014; Zung, 1965). Existing studies have shown that negative emotions can stimulate an individual’s emotional regulation mechanisms and may further promote the generation of procrastination behaviors (Eerde, 2003; Walsh & Ugumba-Agwunobi, 2002). In this context, sleep procrastination is easily associated with negative emotions, such as anxiety and depression. According to the diathesis-stress model of depression, stress is considered an important factor associated with depression (Bleuler, 1963). An individual’s perception of stress has a positive predictive effect on the degree of depression (Nikčević et al., 2014), which means that with an increase in the perceived level of stress, the degree of depression will also increase significantly (Z. Liu et al., 2021). Therefore, it can be speculated that depression may have a mediating role between perceived stress and sleep procrastination.

Inhibitory control refers to an individual’s inhibition of a dominant reaction, habitual behaviors, and/or interference stimuli unrelated to the target task under a specific environment. This psychological mechanism has an important role in the execution of complex tasks, especially as an important internal factor affecting procrastination behaviors. An empirical study clearly revealed that there was a significant negative correlation between procrastination behavior and individual inhibitory control ability (Gustavson et al., 2015). Bedtime procrastination is a specific form of procrastination involving delaying sleep without external justification. A systematic review and meta-analysis involving 43 studies concluded that bedtime procrastination is moderately negatively associated with self-control, which overlaps with inhibitory control (Hill et al., 2022). More specifically, individuals with a high propensity to procrastinate showed a relatively weak state of inhibitory control (Rebetez et al., 2018), further strengthening the negative correlation between self-control and inhibitory control. In addition, other studies have pointed out that when faced with three types of positive, neutral, and negative emotional stimuli, the degree of impaired inhibitory control ability is most significant in negative emotional situations. Specifically, depressive experiences can be associated with weaker ability to inhibit and control negative stimuli (De Raedt & Koster, 2010; Goeleven et al., 2006). Based on these findings, it can be speculated that inhibitory control has a mediating role between depression and sleep procrastination.

Most of the previous studies focused on the basic mechanism underlying procrastination behavior. However, the specific details were not provided. Although some studies have preliminarily explored the correlation between perceived stress and sleep procrastination, these studies have not fully revealed how perceived stress specifically relates to sleep procrastination behavior and the possible mediation mechanism, which undoubtedly limits the effective implementation and precise positioning of relevant intervention measures. In view of this, this study builds a theoretical analysis framework based on the Temporal Self-Regulation Theory (Hall & Fong, 2007) and aims to reveal the status of college students’ sleep procrastination and further explore the mechanism underlying perceived stress affecting sleep procrastination. Based on the theoretical framework outlined above, the following hypotheses are proposed:

**H1.**  *Perceived stress is significantly and positively associated with sleep procrastination among college students.*

**H2.**  *Depression mediates the relationship between perceived stress and sleep procrastination.*

**H3.**  *Inhibitory control mediates the relationship between perceived stress and sleep procrastination.*

**H4.**  *Depression and inhibitory control serve as serial mediators in the relationship between perceived stress and sleep procrastination. Therefore, perceived stress is associated with increased depression, which in turn is associated with impaired inhibitory control and ultimately corresponds to increased sleep procrastination.*

**Figure 1:**
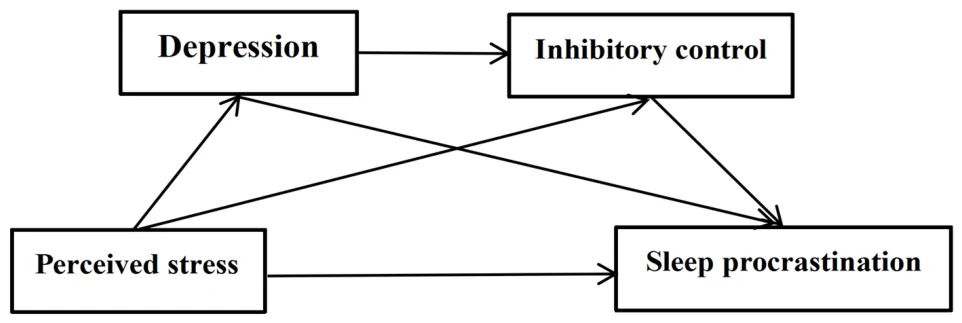
Perceived stress → Sleep procrastination chain mediation model.

The hypothesized serial mediation model is depicted in Figure 1, illustrating the direct path from perceived stress to sleep procrastination, the indirect paths through depression and inhibitory control individually, and the serial mediation path through both mediators sequentially.

Our serial mediation model extends TST by specifying not merely that affective (depression) and self-regulatory (inhibitory control) mechanisms matter, but in what order they operate. Whereas prior studies have treated these as parallel pathways, our findings demonstrate a cascading logic: perceived stress first fosters depressive symptoms, which then impair inhibitory control, and this sequential depletion ultimately links to sleep procrastination. This ordering distinction is theoretically meaningful because it clarifies that emotional distress precedes and constrains self-regulatory capacity—a refinement with direct implications for how interventions are sequenced.

## 2. Materials and Methods

### 2.1. Ethical Approval Statement

This study strictly followed the principles of scientific research ethics, which were formally approved by the Ethics Committee of Harbin Medical University in advance (HMUIRB20200002). All participants completed the questionnaire after acknowledging informed consent.

### 2.2. Participants and Procedure

This study adopted a cluster random sampling method and selected three universities in Heilongjiang Province as sample sources, with a total of 1673 students as research objects from March to April 2024. A total of 1986 questionnaires were distributed to students at these universities during the study. Questionnaires were excluded for any of the following criteria: (1) missing data exceeding 10% of items per scale; (2) extreme response patterns (e.g., identical answers for >90% of items); or (3) completion time < 1 min, which was considered indicative of insufficient attention. After data cleaning, 1673 valid questionnaires were retained, yielding an effective response rate of 84.2%. These 1673 respondents constituted the final study sample.

### 2.3. Measures

The general data questionnaire was designed by the researcher and included gender, family relationship, frequency of communication with parents, stress, and burden during the school year, and average daily sleep duration.

The Bedtime Procrastination Scale (BPS) was designed by Kroese et al. (2014) and consists of nine items. The scoring scale was a Likert 5-point scale ranging from 1 (never) to 5 (always), with four items reverse-scored (Kroese et al., 2014). The BPS was shown to have good internal consistency with an internal consistency coefficient of 0.92.

The Perceived Stress Scale (PSS) was developed by S. Cohen et al. (1983). The scale includes 14 items covering two dimensions. The scoring criteria use a 5-point Likert scale; the higher the score, the greater the perceived stress. In the present study, the Cronbach’s α coefficient for the PSS was 0.78, indicating acceptable internal consistency.

The Patient Health Questionnaire-9 items (PHQ-9) were designed to assess the frequency of depression-related symptoms in an individual during the past 2 weeks. The scale was developed by Kroenke and colleagues (Kroenke et al., 2001) and consists of nine items. The scoring standard adopted the Likert four-level scoring method with 0–3 representing no to almost every day, respectively; the higher the score, the more severe the depression. The internal reliability of the PHQ-9 was very good, with a Cronbach’s α of 0.89.

The Behavior Rating Inventory of Executive Function Adult Version (BRIEF-A), developed by Roth et al. (2005), is a self-report measure designed to assess executive function difficulties in everyday life. The inhibitory control subscale, which was selected for analysis in this study, contains eight items rated on a three-point scale (1 = “never” to 3 = “often”), with higher scores indicating greater difficulties in inhibitory control [i.e., poorer inhibitory control ability] (Gioia et al., 2000). This subscale captured perceived difficulties in resisting impulses, stopping behavior when necessary, and exercising behavioral restraint in daily contexts. The Cronbach’s α coefficient for this subscale in the current sample was 0.67, which is relatively modest but consistent with the broad-band nature of executive function measures in non-clinical populations.

### 2.4. Statistical Methods

SPSS27.0 software was used for data analysis in this study. Descriptive analysis was used to describe the basic characteristics of college students. A *t*-test and ANOVA were used to compare the differences in sleep procrastination using demographic variables. Pearson correlation analysis was used to analyze the correlation between variables. Multiple hierarchical regression analysis was used to determine the effects of perceived stress, depression, and inhibitory control on sleep procrastination. The mediation effect was analyzed using Model 6 in the PROCESS plugin (version 4.2; Hayes, 2022) for SPSS. Bootstrapping with 5000 random samples was performed to evaluate the significance of the mediating effect.

## 3. Results

### 3.1. Demographics

Male students accounted for 56.78% and female students accounted for 43.22% of the participants in this study. Among the college students, 62.34% were not only children and 26.18% had a very harmonious family relationship. The overall mean score of sleep procrastination was 37.2, the median score was 38, and the interquartile range was 36.00–40.00. The sleep procrastination scores were <36 points in 22.89% of the students, indicating a low degree of procrastination. Among the 31.32% of students who scored between 36 and 38 points, the sleep procrastination score was within the middle and low levels. Among the 30.30% of students who scored between 38 and 40 points, the sleep procrastination behavior was close to or had reached a high level. In addition, 15.48% of the students scored >40 points, showing a significant tendency to sleep procrastination. In addition, the sleep procrastination score was statistically significant by gender. The average daily sleep duration and the number of days the students exercised for >30 min in the past 7 days (*p* < 0.05; Table 1).

In summary, the sleep procrastination scores were predominantly at or above the medium level among college students. Significant differences in sleep procrastination were observed across gender, average daily sleep duration, and exercise frequency. Students with shorter sleep duration and those exercising less frequently reported higher levels of sleep procrastination.

### 3.2. Correlation Analysis of Perceived Stress, Depression, Inhibitory Control and Sleep Procrastination Scores

The perceived stress score of college students was 58.24 ± 5.72, the depression score was 18.90 ± 4.70, the inhibitory control score was 12.22 ± 1.93, and the sleep procrastination score was 37.20 ± 3.88. Perceived stress was significantly positively correlated with sleep procrastination (r = 0.596, *p* < 0.01) and depression (r = 0.605, *p* < 0.01), and negatively correlated with inhibitory control (r = −0.520, *p* < 0.01). Depression was positively correlated with sleep procrastination (r = 0.568, *p* < 0.01) and negatively correlated with inhibitory control (r = −0.452, *p* < 0.01). There was a significant negative correlation between inhibitory control and sleep procrastination (r = −0.431, *p* < 0.01; Table 2).

Harman’s single-factor test was performed to assess the potential influence of common method bias. All items from the PSS, PHQ-9, Inhibitory Control subscale, and BPS were entered into an unrotated principal component factor analysis. The results indicated that the first factor accounted for 15.620% of the total variance, which is below the recommended threshold of 40%, suggesting that common method bias does not pose a serious threat to the validity of the findings in this study.

In summary, all study variables demonstrated significant intercorrelations in the expected directions. Perceived stress and depression were positively associated with sleep procrastination, while inhibitory control showed a negative association, providing preliminary support for the hypothesized mediation model.

### 3.3. Multiple Regression Analysis of Factors Influencing Sleep Procrastination Among College Students

A multiple linear stepwise regression analysis was performed to identify the factors influencing sleep procrastination among college students, with sleep procrastination as the dependent variable and perceived stress, depression, and inhibitory control as independent variables. Diagnostics verified the underlying assumptions before interpreting the regression results. Residual plots suggested acceptable normality and homoscedasticity. Pairwise correlations among the predictors were all <0.75. The variance inflation factor (VIF) values ranged from 1.492 to 2.211, all below the threshold of 5, and tolerance values ranged from 0.557 to 0.700, all exceeding 0.2, jointly indicating no substantial multicollinearity. The detailed regression results are presented in Table 3.

In summary, after controlling for demographic variables, perceived stress, depression, and inhibitory control each independently contributed to sleep procrastination, collectively accounting for a substantial portion of the variance. No multicollinearity issues were detected among the predictor variables.

### 3.4. Mediating Role of Depression and Inhibitory Control in the Chain Between Perceived Stress and Sleep Procrastination of College Students

The Model 6 framework in PROCESS software was used to analyze and verify the chain mediation effect. The independent variable was perceived stress and the dependent variable was sleep procrastination. The mediating variables were depression and inhibitory control, and the pathways between perceived stress and sleep procrastination were explored. In addition, gender, average daily sleep duration, and the number of days of exercise for >30 min in the past 7 days were used as control variables to further enhance the accuracy and reliability of the analysis results (Table 4).

**Figure 2:**
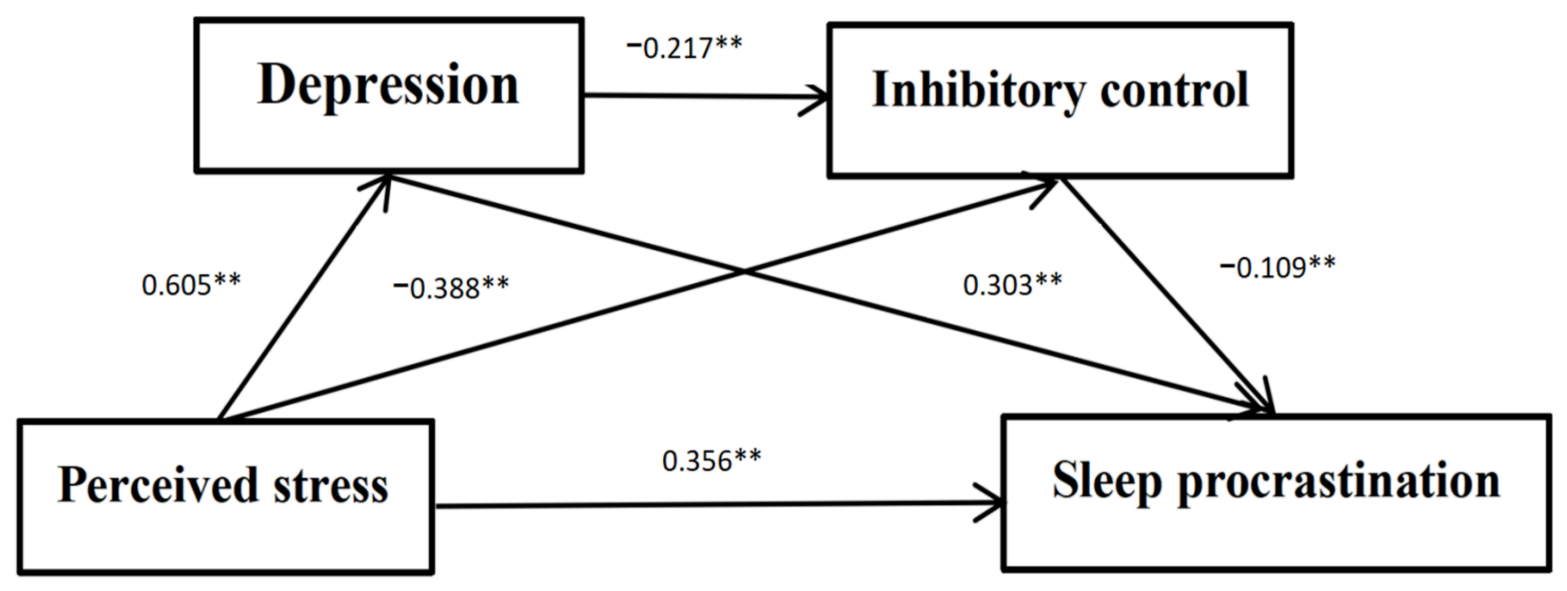
Perceived stress → Sleep procrastination chain mediation model (** *p* < 0.01).

Analysis of the bootstrap test results revealed the significance of indirect effects of the three paths because the 95% confidence interval of these effects did not contain the value 0. As shown in Table 5, the mediation effect analysis showed that depression has a significant mediating role in the relationship between perceived stress and sleep procrastination, and the 95% confidence interval was 0.147–0.215 (Table 5). Inhibitory control had a significant mediating role in the relationship between perceived stress and sleep procrastination, and the 95% confidence interval was 0.021–0.064. Depression and inhibitory control were significant chain mediators in the relationship between perceived stress and sleep procrastination, with 95% confidence intervals of 0.007–0.023 (Table 4 and Figure 2).

In summary, both depression and inhibitory control independently mediated the relationship between perceived stress and sleep procrastination. Critically, the serial mediation pathway through depression followed by inhibitory control was also significant, supporting the hypothesized chain mediation mechanism. The total indirect effect indicated that mediation accounted for a meaningful proportion of the overall relationship.

## 4. Discussion

This study investigated the associations between perceived stress, depression, inhibitory control, and sleep procrastination among college students with a particular focus on the serial mediating roles of depression and inhibitory control. The results revealed a sequential pattern of associations. Specifically, perceived stress was directly associated with sleep procrastination. Moreover, this relationship was indirectly associated through the following: (a) depression alone; (b) inhibitory control alone; and (c) the serial pathway linking depression to inhibitory control.

The sleep procrastination scores in this sample were predominantly distributed at or above the medium level, indicating that delayed bedtime is a widespread phenomenon among college students, which is consistent with previous epidemiologic estimates (Huang et al., 2023). Notably, males, students reporting shorter average daily sleep duration, and students exercising less frequently exhibited higher sleep procrastination scores. These demographic patterns aligned with prior evidence that gender-specific nocturnal activities (e.g., gaming) may extend wakefulness in males (Gandaputra et al., 2021; Tsai & Li, 2004) and regular physical activity may mitigate procrastinatory tendencies by reducing perceived stress and ruminative thinking (D. Liu et al., 2025). However, given the cross-sectional nature of these data, these associations should be interpreted as descriptive correlates rather than causal determinants.

We first examined the direct association between perceived stress and sleep procrastination. The data revealed a positive and significant correlation independent of demographic covariates. This finding is consistent with the resource depletion perspective (Baumeister, 2002; Heatherton & Tice, 1994). From this viewpoint, individuals who report higher levels of perceived stress may be more likely to engage in evening leisure activities, such as watching videos or playing games, as a means of coping with daytime fatigue (Kashani et al., 2012; Nauts et al., 2019). Such behavioral patterns, if sustained, could be interpreted as contributing to a cycle in which delayed sleep onset co-occurs with elevated next-day stress, although the cross-sectional nature of our data precludes confirmation of this temporal sequence.

Beyond this direct association, depression emerged as a significant independent mediator. Perceived stress was positively correlated with depressive symptoms, which in turn were positively correlated with sleep procrastination. This pattern of associations is theoretically consistent with the diathesis-stress model of depression (Bleuler, 1963) and aligns with prior empirical evidence showing that stress perception is a robust correlate of depressive severity (Z. Liu et al., 2021; Nikčević et al., 2014). In the bedtime context, individuals with elevated depressive symptoms may report reduced motivation to adhere to scheduled sleep times and a diminished cognitive appreciation of the restorative functions of sleep. These factors could help explain why depressive symptoms are associated with a greater tendency to intentionally delay bedtime, even when individuals are aware of the potential negative consequences.

A second distinct pathway involved inhibitory control. Perceived stress was negatively correlated with inhibitory control and poorer inhibitory control was correlated with higher sleep procrastination. This pattern of associations is consistent with the ego-depletion model (Baumeister, 2002), which suggests that individuals experiencing higher stress may have fewer self-regulatory resources available to override prepotent responses, such as engaging with smartphones or social media, in favor of the goal of timely sleep. However, as with other pathways discussed above, this interpretation is offered as a theoretical account that awaits confirmation through longitudinal or experimental designs.

In addition to these independent mediating pathways, the serial pattern of associations (perceived stress → depression → inhibitory control → sleep procrastination) was also supported. The serial pathway refines TST by specifying a causal logic the framework implies but has not operationalized: depressive symptoms, as an intention-related affective state, may precede and undermine the executive resources (inhibitory control) required to execute sleep intentions. This moves the field beyond asking whether affect and self-control both matter to asking under what affective conditions self-control becomes compromised. This sequential pattern is consistent with the hopelessness theory of depression (Alloy et al., 1988), which suggests that stressful experiences are associated with depressive cognitive styles that may relate to reduced executive functioning. Importantly, the plausibility of this affective-to-executive sequence is bolstered by emerging neurocognitive evidence. For example, Federico et al. (2022) reported that poorer subjective sleep quality was significantly correlated with depressive symptoms, visuospatial working memory performance, and altered resting-state functional connectivity within the limbic and fronto-temporo-parietal networks. While the present study did not utilize direct neural measures, such neurobiological findings support the conceptual relevance of linking depressive states to inhibitory control in the context of sleep-related decision-making. Our results extend this neurocognitive literature by identifying a behavioral outcome (sleep procrastination) that may co-vary with both affective dysregulation and self-regulatory difficulties. This pattern provides correlational evidence consistent with the Temporal Self-Regulation Theory framework (Hall & Fong, 2007), in which both intention-related (affective) and self-regulatory (executive) components may jointly relate to health behavior.

A comparative examination of the indirect effects revealed meaningful disparities in magnitude. Depression alone accounted for approximately 30.2% of the total effect, inhibitory control alone contributed 7.0%, and the serial pathway contributed 2.3%. These differential effect sizes carry practical implications for intervention design. The dominant contribution of depression suggests that large-scale prevention efforts may most efficiently target depressive symptomatology. For example, through cognitive-behavioral therapy, mindfulness-based stress reduction, or enhanced access to campus counseling services. Nevertheless, the serial pathway, albeit small, identifies a clinically relevant subgroup of students who may present with comorbid emotional distress and executive dysfunction. For these individuals, combined affective-regulatory interventions, such as integrated cognitive remediation and emotion regulation training, may yield incremental benefits. In addition, low-cost behavioral strategies, like structured physical exercise, should not be overlooked because previous work has shown that exercise reduces perceived stress and ruminative thinking, thereby indirectly preserving inhibitory control and sleep quality (D. Liu et al., 2025).

Implications for intervention. The serial pattern yields graded implications. First, depression accounted for the largest indirect effect (30.2%), suggesting that campus-wide screening and early intervention for depressive symptoms may offer the broadest preventive return. Second, the serial pathway, though small (2.3%), identifies a subgroup with co-occurring emotional and regulatory difficulties who may require sequential protocols—addressing depressive rumination first, then reinforcing inhibitory control via implementation intentions or bedtime-specific training. Third, low-threshold strategies such as structured physical exercise, which reduces stress and rumination, could serve as a universal preventive tier. Universities may consider tiered pathways: universal stress reduction, targeted depression counseling, and combined affective–regulatory interventions for students with higher bedtime procrastination.

Several limitations of this study should be acknowledged. First, the cross-sectional design precludes causal inferences regarding the observed associations among perceived stress, depression, inhibitory control, and sleep procrastination. Future research adopting longitudinal or experimental designs is needed to establish the temporal ordering and causal directions of these relationships. Second, all data were collected via self-report questionnaires, which may introduce response biases and social desirability effects. The reliance on self-reported inhibitory control may not fully capture the neurocognitive aspects of this construct. Future studies could incorporate behavioral tasks or neurophysiological measures to provide more objective assessments. Third, the sample was limited to universities in Heilongjiang Province, which may restrict the generalizability of findings to other regions or cultural contexts. Although participants were recruited from three universities, the present study did not account for potential clustering effects at the institutional level. Future studies with larger samples from multiple institutions may employ multilevel modeling to account for such clustering. Fourth, the results obtained (e.g., correlations, differences) may be unstable across different samples, time points, and settings due to the relatively low reliability demonstrated by the BRIEF-A in this study, potentially reducing replicability in future experiments. Fifth, other potentially relevant variables, such as smartphone use before bedtime, chronotype, academic workload, and social media engagement, were not included in the model and may confound the observed relationships. Future research should consider incorporating these factors to develop a more comprehensive understanding of sleep procrastination mechanisms.

## 5. Conclusions

In summary, perceived stress was indirectly associated with higher levels of sleep procrastination in college students with depression, with inhibitory control serving as a serial mediator in this relationship. This process not only reveals the complex relationship between mental health status and behavioral regulation ability but also provides a new perspective and theoretical basis for understanding and intervening in sleep problems in college students. Practically, our findings advocate for tiered and targeted interventions: addressing depressive symptoms as a primary target to interrupt the chain at its source, while concurrently implementing inhibitory control training to fortify self-regulatory capacity against the final step of bedtime delay. These insights offer a more precise theoretical and practical roadmap for developing effective, mechanism-based prevention programs for sleep procrastination in student populations.

## Figures and Tables

**Table 1:** Basic information of students (*n* = 1673).

Variable	Number of People (%)	*M* ± *SD*	*t*/*F*	*p*
**Gender**			2.127	0.034
Male	950 (56.78%)	37.38 ± 3.66		
Female	723 (43.22%)	36.97 ± 4.15		
**Whether is an only child**			1.735	0.083
Yes	630 (37.66%)	37.41 ± 3.75		
No	1043 (62.34%)	37.07 ± 3.96		
**Family relationship**			0.639	0.590
In perfect harmony	334 (19.96%)	37.35 ± 3.86		
Very harmonious	438 (26.18%)	37.10 ± 3.82		
Normal	627 (37.48%)	37.11 ± 3.93		
Disharmony	274 (16.38%)	37.41 ± 3.92		
**Frequency of communication with parents**			1.908	0.126
A lot	271 (16.20%)	36.98 ± 3.77		
More	423 (25.28%)	36.95 ± 3.95		
normal	637 (38.08%)	37.27 ± 4.00		
Very few	342 (20.44%)	37.56 ± 3.65		
**Stress and burden this school year**			1.515	0.195
None	150 (8.96%)	37.09 ± 3.56		
Lesser	278 (16.62%)	36.94 ± 4.21		
Normal	549 (32.82%)	37.02 ± 3.75		
Larger	406 (24.27%)	37.48 ± 3.81		
Maximum	290 (17.33%)	37.46 ± 4.04		
**Average daily sleep duration**			4.053	0.007
More than 8 h	671 (40.11%)	37.13 ± 3.85		
7–8 h	604 (36.10%)	36.96 ± 3.94		
6–7 h	255 (15.24%)	37.42 ± 3.95		
Less than 6 h	143 (8.55%)	38.16 ± 3.58		
**The number of days that you exercised for more than 30 min in the past 7 days**			5.491	0.004
More than 5 days	435 (26.00%)	37.05 ± 3.89		
3–5 days	519 (31.02%)	36.84 ± 4.19		
0–3 days	719 (42.98%)	37.55 ± 3.62		

**Table 2:** Correlation analysis of college students’ perceived stress, depression, inhibitory control and sleep procrastination scores.

Variable	*M* ± *SD*	Perceived Stress	Depression	Inhibitory Control	Sleep Procrastination
Perceived stress	58.24 ± 5.72	1			
Depression	18.90 ± 4.70	0.605 **	1		
Inhibitory control	12.22 ± 1.93	−0.520 **	−0.452 **	1	
Sleep procrastination	37.20 ± 3.88	0.596 **	0.568 **	−0.431 **	1

** *p* < 0.01.

**Table 3:** Multiple linear regression analysis of bedtime procrastination in college students.

	B	Standard Error	β	t	*p*	Tolerance	VIF
constant	21.090	1.220	-	17.292	<0.001	-	-
Perceived stress	0.241	0.017	0.356	14.397	<0.001	0.557	2.211
Depression	0.251	0.020	0.303	12.807	<0.001	0.607	1.944
Inhibitory control	−0.220	0.044	−0.109	−4.958	<0.001	0.700	1.492

**Table 4:** Hierarchical regression analysis of mediating effects of perceived stress on sleep procrastination.

Influencing Factors	Step One		Step Two		Step Three	
	Dependent Variables: Depression		Dependent Variables: Inhibitory Control		Dependent Variables: Sleep Procrastination	
	β	t	β	t	β	t
Perceived stress	0.605	31.101 **	−0.388	−15.078 **	0.356	14.397 **
Depression			−0.217	−8.446	0.303	12.807 **
Inhibitory control					−0.109	−4.958 **
R^2^	0.367		0.300		0.432	
F	967.267 **		357.522 **		422.474 **	

** *p* < 0.01.

**Table 5:** Analysis of the pathway effect of perceived stress on sleep procrastination.

	Effect	BootSE	95%CI
X → M1 → Y	0.180	0.017	[0.147, 0.215]
X → M2 → Y	0.042	0.011	[0.021, 0.064]
X → M1 → M2 → Y	0.014	0.004	[0.007, 0.023]

X: perceived stress; Y: sleep procrastination; M1: depression; M2: inhibitory control.

## Data Availability

The data presented in this study are available on reasonable request from the corresponding author.
